# Supplementary material for: Refining epileptogenic high-frequency oscillations using deep learning: a reverse engineering approach
Source: Brain Commun. 2021 Nov 3;4(1):fcab267. doi: 10.1093/braincomms/fcab267 (PMC8833577; doi:10.1093/braincomms/fcab267)
Supplement: fcab267_Supplementary_Data [file fcab267_supplementary_data.zip › Supplementary_table.docx]

| **Supplementary Table** | |  |  |
| --- | --- | --- | --- |
|  |  |  |  |
|  | Mean pixel statistical value at the HFO event onset (-45ms to +45 ms) | |  |
| Pt. No. | Ripples | Fast ripples |  |
| 1 | 0.35 | 0.57 |  |
| 2 | 0.82 | 0.41 |  |
| 3 | 0.41 | 0.50 |  |
| 4 | 0.00 | 0.00 |  |
| 5 | 0.85 | 0.50 |  |
| 6 | 0.76 | 0.28 |  |
| 7 | 0.33 | 0.03 |  |
| 9 | 0.00 | 0.17 |  |
| 10 | 0.68 | 0.67 |  |
| 11 | 0.04 | 0.00 |  |
| 12 | 0.71 | 0.39 |  |
| 13 | 0.00 | 0.00 |  |
| 15 | 0.77 | 0.22 |  |
| 16 | 0.96 | 0.86 |  |
| 17 | 0.50 | 0.26 |  |
| Overall | 0.48 | 0.32 | *p = 0.15 |
|  |  |  |  |
| *Two-tailed t-test | |  |  |
