# Supplementary figures and images for: Refining epileptogenic high-frequency oscillations using deep learning: a reverse engineering approach
Source: Brain Commun. 2021 Nov 3;4(1):fcab267. doi: 10.1093/braincomms/fcab267 (PMC8833577; doi:10.1093/braincomms/fcab267)

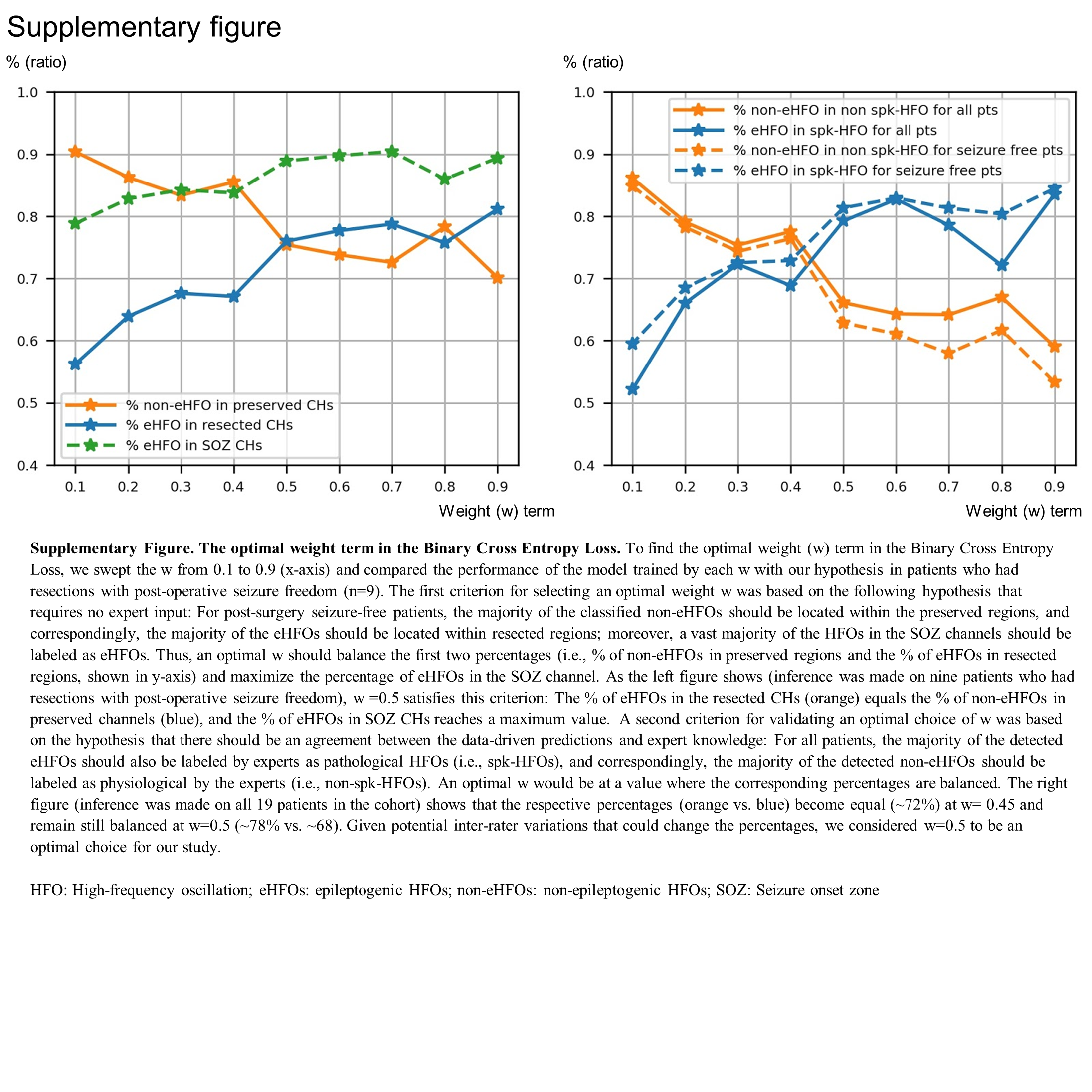

Supplement: fcab267_Supplementary_Data [file fcab267_supplementary_data.zip › Supplementary_figure.tif]
